# Natural Product Interventions for Chemotherapy and Radiotherapy-Induced Side Effects

**DOI:** 10.3389/fphar.2018.01253

**Published:** 2018-11-06

**Authors:** Qing-Yu Zhang, Fei-Xuan Wang, Ke-Ke Jia, Ling-Dong Kong

**Affiliations:** ^1^School of Medicine and Life Sciences, Nanjing University of Chinese Medicine, Nanjing, China; ^2^Department of Pathology, Sir Run Run Hospital, Nanjing Medical University, Nanjing, China; ^3^State Key Laboratory of Pharmaceutical Biotechnology, School of Life Sciences, Nanjing University, Nanjing, China

**Keywords:** natural products, cancer, chemotherapy, radiotherapy, side effects

## Abstract

Cancer is the second leading cause of death in the world. Chemotherapy and radiotherapy are the common cancer treatments. However, the development of adverse effects resulting from chemotherapy and radiotherapy hinders the clinical use, and negatively reduces the quality of life in cancer patients. Natural products including crude extracts, bioactive components-enriched fractions and pure compounds prepared from herbs as well as herbal formulas have been proved to prevent and treat cancer. Of significant interest, some natural products can reduce chemotherapy and radiotherapy-induced oral mucositis, gastrointestinal toxicity, hepatotoxicity, nephrotoxicity, hematopoietic system injury, cardiotoxicity, and neurotoxicity. This review focuses in detail on the effectiveness of these natural products, and describes the possible mechanisms of the actions in reducing chemotherapy and radiotherapy-induced side effects. Recent advances in the efficacy of natural dietary supplements to counteract these side effects are highlighted. In addition, we draw particular attention to gut microbiotan in the context of prebiotic potential of natural products for the protection against cancer therapy-induced toxicities. We conclude that some natural products are potential therapeutic perspective for the prevention and treatment of chemotherapy and radiotherapy-induced side effects. Further studies are required to validate the efficacy of natural products in cancer patients, and elucidate potential underlying mechanisms.

## Introduction

Cancer is one of the most common causes of death worldwide (Mcguire, 2016). Chemotherapy and radiotherapy are the most effective and extensive approaches for cancer management. However, chemotherapy, and radiotherapy-induced adverse effects including oral mucositis, gastrointestinal toxicity, hepatotoxicity, nephrotoxicity, hematopoietic system injury, cardiotoxicity, and neurotoxicity, hinder the clinical uses. Subsequently, these harmful effects often reduce the quality of life in cancer patients, and may lead to therapy discontinuation (Shapiro, 2016; Turcotte et al., 2017). Therefore, it is important to develop effective management strategies against chemotherapy and radiotherapy-induced side effects.

Natural products including crude extracts, bioactive components-enriched fractions, and pure compounds derived from herbs as well as herbal formulas have been proved to prevent and treat cancers (Sanders et al., 2016). Clinical trials and preclinical studies show that some natural products can reduce chemotherapy and radiotherapy-induced oral mucositis, gastrointestinal toxicity, hepatotoxicity, nephrotoxicity, hematopoietic system injury, cardiotoxicity, and neurotoxicity. Natural dietary supplements containing some ingredients such as Ginseng extract, Grape seed extract, and curcumin promote the recovery from severe illness, and relieve chemotherapy and radiotherapy-induced side effects. Gut microbiota plays the role in the modulation of drug action (Curro, 2018), possibly could evaluate the risk of severe gastrointestinal toxicity. Herb medicine has potential to improve digestive health (Peterson et al., 2018). The aim of this review is to summarize the recent evidence in detail for the effectiveness of natural products, and describe the possible mechanisms of the actions in reducing chemotherapy and radiotherapy-induced side effects. This review also draws particular attention to recent advances in the efficacy of natural dietary supplements, and the modulation of natural products on gut microbiotan for the protection against cancer therapy-induced toxicity.

## Prevention of oral mucositis partly by reducing oxidative stress, inflammation, and infection

Oral mucositis is a common complication in patients undergoing chemotherapy and radiotherapy, which is initiated partly by oxidative stress and inflammation. Plant-derived and other natural medicines with anti-inflammation are recently demonstrated to manage chemotherapy-induced oral mucositis (Panahi et al., 2016; Mahendran et al., 2018). *Plantago major* is a most abundant herbaceous perennial plant that has been traditionally used for hundreds of years, and is effective as a wound healer with anti-ulcerative, anti-inflammatory, anti-bacterial, anti-viral, and anti-oxidant activities (Adom et al., 2017). Moreover, its extract is reported to have anticancer effect in Ehrlich ascites tumor bearing Balb/C mice (Ozaslan et al., 2007). A feasibility randomized triple-blind phase III clinical trial shows that *P. major* extract, chlorhexidine 0.12% and sodium bicarbonate 5% solution can effectively manage oral mucositis in cancer patients with grade II-III mucositis (Cabrera-Jaime et al., 2018).

Severe stomatitis may interrupt or discontinue cancer therapeutics, and increase the risk of local and systemic infection. Japanese traditional medicine (Kampo) Hangeshashinto (TJ-14) effectively relieves chemotherapy-induced oral mucositis in gastric cancer and colorectal cancer patients by anti-oxidation and anti-inflammation (Matsumoto et al., 2015; Nishikawa et al., 2018) or suppression of inflammatory cell chemotaxis and cyclooxygenase-2 (COX2) expression (Kamide et al., 2017). Chamomile (*Matricaria recutita*) is a medicinal plant widely used in traditional medicine for its anti-oxidant, anti-microbial, and anti-inflammatory actions (Gomes et al., 2018), which seems to be a promising alternative for the treatment of 5-fluorouracil-induced oral mucositis and recurrent aphthous stomatitis (Seyyedi et al., 2014; Gomes et al., 2018). Aznol mouthwash combined with chamomile extract and Hangeshashinto, alleviates stomatitis in a small cohort of lung cancer patients treated with afatinib (Kato et al., 2017). *Aloe vera*, an herbal medicine in tropical area, has been used to treat skin disorders with anti-bacterial and wound-healing effects. Its mouthwash significantly reduces the intensity of chemotherapy-induced stomatitis and pain in clients with lymphoma and leukemia, and possibly improves the nutritional status of cancer patients (Mansouri et al., 2016).

Natural nutritional products are also investigated to reduce chemotherapy and radiotherapy-induced oral mucositis. Turmeric (the rhizome of *Curcuma longa*) is a traditional spice and herb with many health benefits. Turmeric and its active ingredient curcumin have anti-oxidant and anti-inflammatory properties. Anticancer effects of curcumin have been demonstrated in a number of preclinical studies as well as in phase I/II clinical trials (Hatcher et al., 2008). Curcumin mouthwash is observed to be better than chlorhexidine mouthwash in terms of rapid wound healing in adult patients with chemotherapy and radiotherapy-induced oral mucositis (Patil et al., 2015). The rhizome of *Zingiber officinale* (ginger) is also used as traditional medicine with health benefits to treat muscular aches and pains worldwide. Its main ingredients 6-gingerol and 6-shogaol are reported to relief 5-fluorouracil-induced oral ulcerative mucositis and pain by regulating Na^+^ channels, playing an essential role in ginger-associated analgesia of pain (Hitomi et al., 2017). Tocotrienols are natural compounds existed in vegetable oil, which are a promising analogs of vitamin E for cancer therapeutics by sensitizing cancer cells to chemotherapeutic agents (Sailo et al., 2018). γ-Tocotrienol can prevent 5-fluorouracil-induced reactive oxygen species (ROS) generation in human oral keratinocytes by stabilizing the activation of nuclear factor erythroid 2-related factor 2 (Nrf2), a redox-sensitive master regulatory transcription factor (Takano et al., 2015). Quercetin, a natural flavonoid enriched in common vegetables and herbs, has anti-oxidative and anti-inflammatory effects. Administered 250 mg quercetin capsules twice daily for 4 weeks, can reduce the incidence of oral mucositis in patients who undergo high dose chemotherapy for blood malignancy (Kooshyar et al., 2017).

Honey produced by bees from flower nectar, is a folk medicine since ancient times in many countries. A meta-analysis indicates that honey can effectively reduce the incidence of chemotherapy and radiotherapy-induced oral mucositis (Xu et al., 2016). Further clinical evidence demonstrates its effectiveness as a preventative and therapeutic measure for chemotherapy-associated oral mucositis in pediatric oncology patients (Friend et al., 2018). There is potential interaction between oral microenvironment and mucositis development. Honey treatment produces the significant reduction of oral mucositis associated *Candida* and aerobic pathogenic bacterial infection, and increases body weight, delays the onset of oral mucositis, and decreases the severity of pain (Al Jaouni et al., 2017). In Turkey, black mulberry molasses is a foodstuff widely used as traditional intervention for the treatment of mucositis. Recently, black mulberry molasses usage is demonstrated to be an effective intervention in the prevention of radiation-induced oral mucositis in head and neck cancer patients (Demir Dogan et al., 2017). Propolis is a natural product collected by honeybee worker, and contains flavonoids with anti-ulcer, anti-bacterial, anti-fungal, healing and anti-inflammatory effects (Dodwad and Kukreja, 2011). Its water extract mouthwash efficiently prevents and heals chemotherapy or radiotherapy-induced oral mucositis in patients with head and neck cancers, or leukemia (Javadzadeh Bolouri et al., 2015; Eslami et al., 2016). However, early report of a double blind randomized placebo controlled trial shows that propolis cannot be recommended for severe oral mucositis in chemotherapy-treated pediatric patients (Tomazevic and Jazbec, 2013). The natural products in this part together with relevant characteristics of the respective studies are summarized in Table 1.

**Table 1:** Natural products in reducing chemotherapy and radiotherapy-induced oral mucositis.

**Name**	**Effect/Mechanism**	**Experimental setting/Model**	**Ingredients/Source**	**References**
*P. major*	Anti-inflammation	Patients	A variety of bioactive compounds including flavonoids, alkaloids, terpenoids, caffeic acid derivatives, and iridoid glycosides	Cabrera-Jaime et al., 2018
Hangeshashinto	Anti-oxidation and anti-inflammation	Patients	Made from Pinelliae tuber, Scutellariae radix, Glycyrrhizae radix, Zizyphi fructus, Ginseng radix, Zingiberis Processum rhizoma, and Coptidis rhizome Main chemical constituents: baicalin, baicalein, wogonin, acteoside, berberine chloride, coptisine, [6]-shogaol, [6]-gingerol, liquiritin, glycyrrhizic acid, ginsenoside Rg1, ginsenoside Rb1, corymboside, and homogentisic acid	Matsumoto et al., 2015; Kamide et al., 2017; Nishikawa et al., 2018
Chamomile	Anti-oxidation, anti-microbial, and anti-inflammation	Patients	Terpenoids, favonoids, coumarins, alkaloids, polysaccharides, and glycoside derivatives	Seyyedi et al., 2014; Gomes et al., 2018
*A. vera*	Improvement of the nutritional status	Patients	Oleic acid, tannin, saponin, flavonoids, and terpenoids	Mansouri et al., 2016
Curcumin	Anti-oxidation, anti-inflammation and anti-cancer	Patients	Bioactive constituent of Curcuma longa L.	Patil et al., 2015
Ginger	Regulation of Na^+^ channels	Patients	6-gingerol and 6-shogaol	Hitomi et al., 2017
γ-Tocotrienols	Prevention of ROS generation by stabilizing Nrf2 activation	Patients	Analogues of the vitamin E	Takano et al., 2015
Quercetin	Anti-oxidation and anti-inflammation	Patients	Rich in daily vegetables and herbs	Kooshyar et al., 2017
Honey	Reduction of oral mucositis associated *Candida* and aerobic pathogenic bacterial infection	Patients	Glucose, fructose, acids, proteins, minerals, and polyphenols	Xu et al., 2016; Al Jaouni et al., 2017; Friend et al., 2018
Black mulberry molasses	Antimicrobial	Patients	Phenolics and fatty acids	Demir Dogan et al., 2017
Propolis	Not be recommended for severe oral mucositis	Patients	A natural product collected by honeybee worker	Tomazevic and Jazbec, 2013; Javadzadeh Bolouri et al., 2015; Eslami et al., 2016

## Mitigation of gastrointestinal toxicity by regulating gastrointestinal function and gut microbiota

The most common gastrointestinal toxicities induced by chemotherapy and radiotherapy include nausea, vomiting and diarrhea. In 2016, Chen et al. conduct a systematic review and meta-analysis (from 2005 to 2013) and show that *Atractylodes macrocephala, Poria cocos, Coix lacryma-jobi, Astragalus membranaceus, Glycyrrhiza uralensis*, and *Panax ginseng* can significantly reduce oxaliplatin-induced nausea and vomiting in colorectal cancer (Chen M. H. et al., 2016). *Bacopa monnieri* is used in several traditional systems of medicines for the management of epilepsy, depression, and neuropathic pain. Its bacoside-rich n-butanol fraction antagonizes cisplatin-induced retching and vomiting response (Ullah et al., 2017). Red Ginseng, a product with a steaming and drying process of the fresh ginseng, contains the majority of ginsenosides. Its anticancer mechanisms mainly include cell cycle arrest, apoptosis/paraptosis induction, and angiogenesis inhibition (Wang et al., 2016). Recently, a randomized double blind placebo-controlled trial demonstrates that Red Ginseng decreases symptom of nausea, vomiting, dyspnea and fatigue, and its efficacy and safety can improve life quality in patients receiving six cycles of adjuvant taxane- and platinum-based chemotherapy after cytoreductive surgery (Kim H. S. et al., 2017). A phase II randomized double-blind placebo-controlled study shows that safe and well-tolerated 6-gingerol improves the overall complete response rate in nausea and vomiting, appetite as well as quality of life in cancer patients with surgical resection of primary tumor and moderately to highly emetogenic adjuvant chemotherapy (Konmun et al., 2017), further demonstrating that ginger supplementation can significantly reduce the severity of chemotherapy-induced nausea in adult cancer patients (Ryan et al., 2012). *Rhus coriaria*, a flowering shrub, possesses anticancer effect against human colorectal cancer cells and triple-negative breast cancer cells (El Hasasna et al., 2015, 2016; Athamneh et al., 2017). Its resin extract also attenuates angiogenesis and is used in treatment and management of cancer in Kenya (Ochwang'i et al., 2014; Mirian et al., 2015). *Bunium persicum* is a perennial plant in Iran. A randomized, double blind, crossover clinical trial shows that Persumac, a combination of *R. coriaria* and *B. persicum*, may be helpful in patients with refractory chemotherapy**-**induced nausea and vomiting (Nazari et al., 2017).

Huang-Qin decoction is first described in Chinese canonical medicine about 1800 years ago for the treatment of different gastrointestinal symptoms, including nausea, vomiting and diarrhea. This decoction attenuates irinotecan-induced gastrointestinal toxicity and diarrhea in rats possibly via regulating glycine, serine and threonine pathway as well as bile acid metabolism homeostasis. *Scutelleria baicalensis* may play a crucial role in the therapeutic effect of this decoction on irinotecan-induced diarrhea (Cui et al., 2017; Wang X. et al., 2017).

Of note, nutrient absorption and digestion of gastrointestinal tract may be compromised during chemotherapy and radiotherapy-induced damage to non-cancerous gastrointestinal mucosa. In 2018, Mahendran et al. review and show that plant-derived and some natural medicines with anti-inflammation manage chemotherapy-caused gastrointestinal mucositis (Mahendran et al., 2018). Other animal experiments confirm the efficacy of herbal medicines on intestinal mucositis. Wei-Chang-An pill, a traditional Chinese pharmaceutical preparation, possesses anti-inflammatory advantage and gastrointestinal regulation in digestive diseases. This pill promotes the recovery of intestinal function in 5-fluorouracil-induced intestinal mucositis in mice via relieving severe diarrhea and inhibiting gastric emptying and gastrointestinal transit in intestinal homeostasis (Chen Y. et al., 2016). Bu-Zhong-Yi-Qi decoction, a Chinese traditional herbal medicine, is widely used in Asia as an alternative treatment to reduce side effects of chemotherapy. This decoction protects against 5-fluorouracil-induced intestinal mucositis in mice (Gou et al., 2016). Saireito (TJ-114) is often used to treat inflammatory diseases such rheumatoid arthritis, systemic lupus erythematodes, and nephrotic syndrome. TJ-114 can reduce 5-fluorouracil-induced intestinal mucositis in mice by inhibiting cytokine-mediated apoptosis in intestinal crypt cells (Kato et al., 2015). The root of *Rehmannia glutinosa* is one of the commonly used Chinese herbal medicines. Its steamed products alleviate methotrexate-induced rat gut mucositis by inhibiting oxidative stress and inflammation (Shi et al., 2016). Licorice (*G. uralensis*) is reported to possess anti-tumor activity, and effectively treats peptic ulcer. Its pentacyclic triterpenoid derivative 18β-glycyrrhetinic acid has anti-inflammatory, hepatoprotective and anti-oxidant effects. This compound prevents against cisplatin-induced oxidative intestinal damage in rats possibly by inhibiting nuclear factor-kappa B (NF-κB) and caspases (Rashid et al., 2017). Rutin with free radical scavenging and anti-inflammatory actions also significantly protects against methotrexate-induced intestinal lesion damage in rats (Gautam et al., 2016). *Nigella sativa* seed is a traditional herbal medicine throughout the world. Its oil is beneficial in a wide range of gastrointestinal disorders, and significantly attenuates cisplatin-induced alteration of brush border membrane enzymes, carbohydrate metabolism and antioxidant system in the intestine of rats (Shahid et al., 2017b). Thymoquinone, a major principle active ingredient derived from this seed oil, improves gastrointestinal function as well as the redox and metabolic status of intestinal mucosal tissue in rat model of cisplatin-induced intestinal injury (Shahid et al., 2017a).

Radiotherapy-induced esophagitis and enteropathy are the major issues for long-term cancer survivors (Kim J. S. et al., 2017). Chinese herbal medicine Zhu-Ye-Shi-Gao granule can treat recurrent oral ulcer and chronic pharyngitis. A randomized controlled trial shows that this granule decreases the incidence and grade of acute radiation-induced esophagitis, demonstrating its efficacy and safety in patients with lung, esophagus or mediastinal cancer (Wang L. J. et al., 2017). *A. vera* also effectively treats acute radiation proctitis in patients after external-beam radiation therapy of pelvic malignancy (Sahebnasagh et al., 2017). Oroxylin is a natural flavonoid isolated from *S. baicalensis*, and shows its anticancer effect on human primary hepatocellular carcinoma cells and patient-derived tumor xenograft model for hepatoma (Wei et al., 2017). This compound could be a promising radiosensitizer by inducing G2/M phase and activating cell apoptosis in esophageal squamous cell carcinoma exposed to radiotherapy (Tan et al., 2017). Grape seed extracts and its proanthocyanidins are commonly utilized as dietary supplements for anti-oxidant property in cancer patients (Bagchi et al., 2014). A systematic review of preclinical studies shows that grape seed extracts may alleviate chemotherapy and radiotherapy-induced toxicity (Olaku et al., 2015). Grape seed proanthocyanidins have radioprotective action (Katiyar et al., 2017; Yang et al., 2017). Green tea contains a high content of catechins. Compared with placebo group, its tablet shows less frequency and severity of radiotherapy-induced diarrhea but not vomiting in patients with abdomen and pelvic malignancy after receiving radiotherapy (Emami et al., 2014). Therefore, an extract mixture of green tea and grape seed is considered to be a good radioprotector and immune modulator, indicating its possible use as an adjuvant during radiotherapy (El-Desouky et al., 2017).

Silymarin, a complex of flavonolignans extracted from milk thistle (*Silybum marianum*), has been used for centuries as the treatment of different diseases with anti-oxidant and anti-inflammatory activities. Its major constituent silibinin is reported to radiosensitize prostate cancer by inhibiting DNA repair signaling (Nambiar et al., 2015) and improving the response to radiotherapy in invasive bladder cancer (Prack Mc Cormick et al., 2018). *Nephelium lappaceum* is an importantly commercial crop with nutrients and bioactive constituents. Its hydrolysable polyphenol geraniin possesses good anti-oxidant property, and protects intestinal crypt cells from γ-irradiation-induced cell death in mice by suppressing DNA damage, being considered as a radioprotective agent (Bing et al., 2013). *Clerodendrum infortunatum* rich in flavonoids and saponins is used in Ayurvedic and Siddha traditional medicine. Its hydro-alcoholic extract reduces radiation-induced damage to intestinal crypt cells and suppress BCL2-associated X protein (Bax)/B-cell lymphoma (Bcl-2) ratio, DNA repair gene ataxia telangiectasia mutated (ATM) and COX-2 gene in mice exposed to whole-body γ-radiation (Chacko et al., 2017).

The gut microbiota is a vast community of synergistic bacterial species providing health benefits to the host (Westfall et al., 2018). Gastrointestinal tract forms the targets of chemotherapy and radiotherapy-induced toxicity, causing imbalance in the gut microbiota (dysbiosis) (Serban, 2014; Wardill and Tissing, 2017; Bruneau et al., 2018; Curro, 2018). Fucoidans, a group of certain fucose-containing sulfated polysaccharides, are naturally occurring components of certain edible seaweeds and echinoderms in Japan and Korea. Fucoidan intervention can relieve cyclophosphamide-induced intestinal mucosal injury by decreasing inflammation and increasing tight junction protein expression in mice. Low molecular weight of fucoidan (50 kDa) remarkably increases the abundance of short chain fatty acids (SCFAs) producer *Coprococcus, Rikenella*, and *Butyricicoccus* species within the intestinal mucosa compared with the cyclophosphamide group, possibly as an effective supplement to protect against intestinal mucosal barrier damage during chemotherapy via gut microbiota (Shi et al., 2017). PHY906 is derived from Huang-Qin-Tang, as a modulator of irinotecan-based chemotherapy in patients with advanced colorectal cancer (Kummar et al., 2011). Of note, intestinal bacteria can metabolize irinotecan and PHY906. In murine Colon-38 tumor-bearing mice, alteration in the population of intestinal bacteria can not affect the ability of PHY906 to enhance irinotecan anti-tumor activity or reduce irinotecan-induced intestinal toxicity, indicating that the major species of intestinal bacteria may not appear to have a role in the enhancement of the therapeutic index of irinotecan in tumor-bearing mice treated with PHY906 (Lam et al., 2014). SN-38-glucuronide is an active metabolite of irinotecan hydrochloride to be responsible for gastrointestinal toxicity. Interestingly, baicalin, a main constituent in PHY906, is found to reduce intestinal transporter organic anion transporting polypeptide (OATP)1B1-mediated uptake of SN-38, showing its ability to prevent gastrointestinal toxicity (Fujita et al., 2016). Sheng-Jiang-Xie-Xin decoction, a classic traditional Chinese medical formula in *Shang Han Lun*, has been applied to treat gastroenteritis, ulcerative colitis, and diarrheais, as well as irinotecan-induced gastrointestinal toxicity. Recently, this decoction is reported to alter the pharmacokinetics of irinotecan and its metabolites (SN-38 and SN-38G) to alleviate irinotecan-induced diarrhea in rats, partly by altering carboxylesterase 2 (CES2) and jejunal uridine diphosphate-glucuronosyltransferase 1A1(UGT1A1) activity (Guan et al., 2017). These observations indicate that cancer patients with different gut microbiota profiles may benefit from natural product treatment alongside chemotherapy and radiotherapy. The natural products in this part together with relevant characteristics of the respective studies are summarized in Table 2.

**Table 2:** Natural products in reducing chemotherapy and radiotherapy-induced gastrointestinal toxicity.

**Name**	**Effect/Mechanism**	**Experimental setting/Model**	**Ingredients/Source**	**References**
*B. monnieri*	Prevention of increases in dopamine in the brainstem and increases in 5-HT in the intestine	Cisplatin-induced emesis in Suncus murinus	Bacoside II, bacoside A3, bacosaponin C, and isomer of bacosaponin C	Ullah et al., 2017
Red Ginseng	Anti-inflammation	Patients	Ginsenosides	Kim H. S. et al., 2017
6-Gingerol	Anti-emetic activity by inhibiting neurokinin-1, serotonin, and dopamine receptors	Patients	A major component of ginger	Konmun et al., 2017
Persumac	Unknown	Patients	*R. Coriaria* and *B. Persicum*	Nazari et al., 2017
Huang-Qin decoction	Regulation of glycine, serine, and threonine pathway as well as bile acid metabolism homeostasis	Irinotecan-induced gastrointestinal toxicity and diarrhea in rats	Made from *Glycyrrhiza uralensis, Paeonia lactiflora, Scutelleria baicalensis*, and *Ziziphus jujuba* Main chemical constituents: baicalin, wogonoside, oroxylin-A-glucoside, baicalein, wogonin, orxylin-A, paeoniflorin, glycyrrhizic acid, glycyrrhetinic acid, liquiritin, isoliquirition, liquiritigenin, isoliquiritigenin, and ononin	Cui et al., 2017; Wang X. et al., 2017
Wei-Chang-An pill	Anti-inflammation and gastrointestinal regulation	5-fluorouracil-induced intestinal mucositis in mice	Made from the extracts of *Radix aucklandiae, Moschus, Lignum santali, Lignum aquilariae resinatum, Cinnabaris, Cortex magnoliae officinalis, Fructus aurantii, Radix et rhizoma rhei, Pulvis crotonis tiglium, Rhizoma chuanxiong*, and *Fructus jujubae* Main chemical constituents: costunolide, dehydrodehydrocostus lactone, naringin, hesperidin, neohesperidin, magnolol, honokiol, aloe-emodin, rhein, emodin, chrysophanol, and physcion	Chen Y. et al., 2016
Bu-Zhong-Yi-Qi decoction	Reducion of apoptosis and necrosis in intestinal mucosal epithelia via the suppression of inflammatory cytokine upregulation	5-fluorouracil-induced intestinal mucositis in mice	Made from*Radix Astragalus membranaeus, Radix Panax ginseng, Radix Angelica sinensis, Glycyrrhiza uralensis, Cimicifuga foetida Herba Bupleurumchinense, Citrus sinensis Osbeck*, and *Atractylodes macrocephala* Main chemical constituents: astragalosides, acylsucrose derives, dihydroxyflavone-glucopyranoside, liquiritin, liquiritin apioside, neolicuroside, hesperidin, trihydrohyflavone-diglucopyranoside and cimicifugic acid F	Gou et al., 2016
Saireito (TJ-114)	Inhibition of cytokine-mediated apoptosis in intestinal crypt cells	5-fluorouracil-induced intestinal mucositis in mice	A combined formulation of shosaikoto and goreisan containing saikosaponin, ginsenoside, glycyrrhizin, gingerol, and shogaol	Kato et al., 2015
*R. glutinosa*	Inhibition of oxidative stress and inflammation	Methotrexate-induced gut mucositis in rats	Iridoid glycosides, phenethylalcohol glycosides and furfural derivatives	Shi et al., 2016
18β-Glycyrrhetinic acid	Inhibition of nuclear NF-κB and caspases	Cisplatin-induced oxidative intestinal damage in rats	A pentacyclic triterpenoid derivative obtained from the herb liquorice	Rashid et al., 2017
Rutin	Anti-oxidation and anti-inflammation	Methotrexate-induced intestinal lesion damage in rats	A flavone glycoside extensively found in black tea, apple skin peel and buckwheat	Gautam et al., 2016
*N. sativa* oil	Anti-oxidation	Cisplatin-induced intestinal damage in rats	Rich in polyunsaturated fatty acids, such as omega-3 and omega-6 fatty acids, phytosterols and several other substances including thymoquinone (up to 25% in volatile oil), carvacrol, t-anethole, sesquiterpenelongifolene, and 4 terpinol	Shahid et al., 2017b
Thymoquinone	Anti-oxidation	Cisplatin-induced intestinal injury in rats	A major principle active ingredient derived from *N. sativa* oil	Shahid et al., 2017a
Zhu-Ye-Shi-Gao granule	Inhibition of the release of inflammatory cytokines such as IL-1β, IL-8, and tumor necrosis factor-α	Patients with lung, esophagus or mediastinal cancer	Made from Bamboo leaves, Gypsum fibrosuum, Ginseng, Ophiopogon japonicas, Pinellia ternate, Glycyrrhiza uralensis, and Oryza Main chemical constituents: sativa Ginsenoside, ruscogenin, succinic acid, and glycyrrhizic acid	Wang L. J. et al., 2017
*A. vera*	Anti-inflammation	Patients	Acetylated mannans, polymannans, anthraquinone C-glycosides, anthrones, emodin, and various lectins	Sahebnasagh et al., 2017
Oroxylin	Induction of G2/M phase and activation of cell apoptosis	TE13 and ECA109 cells	A natural flavonoid isolated from *S. baicalensis*	Tan et al., 2017
Proanthocyanidin	Repair of damaged DNA-dependent activation of immune sensitivity; amelioration of mitochondrial dysfunction	Irradiation-treated HFL1 cells	A class of polyphenols	Katiyar et al., 2017; Yang et al., 2017
Green tea	Anti-oxidation, anti-bacterial, anti-inflammation, and anti-intestinal motility	Patients with abdomen and pelvic malignancy	Catechins	Emami et al., 2014
Silibinin	Inhibition of DNA repair signaling and improvement of the response to radiotherapy	Irradiation-treated Human prostate carcinoma DU145, PC-3, and 22RV1 cells, mouse keratinocyte JB6 cells, human lung cancer A549 cells, murine non-invasive MB49 and MB49-I cells	A major active constituent of silymarin, a complex of flavonolignans extracted from milk thistle	Nambiar et al., 2015; Prack Mc Cormick et al., 2018
Geraniin	Suppression of DNA damage	Irinotecan treated rats	A hydrolysable polyphenol from *N. lappaceum*	Bing et al., 2013
*C. infortunatum*	Suppression of Bax/Bcl-2 ratio and COX-2 gene, upregulation of DNA repair gene ATM	Mice exposed to whole-body gamma radiation	Flavonoids and saponins	Chacko et al., 2017
Fucoidan	Upregulation of the abundance of SCFA producer Coprococcus, Rikenella, and *Butyricicoccus* species within the intestinal mucosa	Cyclophosphamide-treated mice	Fucose-containing sulfated polysaccharides	Shi et al., 2017
PHY906	Anti-inflammation and anti-cancer	CPT-11 treated tumor-bearing mice	*Glycyrrhiza uralensis, Paeonia lactiflora, Scutelleria baicalensis*, and *Ziziphus jujuba*	Lam et al., 2014
Baicalin	Down-regulation of OATP2B1	CPT-11 treated mice	A main constituent in PHY906	Fujita et al., 2016
Sheng-Jiang-Xie-Xin decoction	Alteration of the activity of CES2 and jejunal UGT1A1	Irinotecan treated rats	Made from *Pinellia ternata, Glycyrrhiza uralensis, Coptis chinensis, Ziziphus jujuba, Zingiber officinale, Scutellaria baicalensis, Codonopsis pilosula*, and *Zingiber recens* Main chemical constituents: 6-gingerol, baicalin, baicalein, wogonin, epiberberine, trigonelline, liquiritin, lobetyolin, rutin, oleanolic acid, betulinic acid, ursolic acid, berberine, and palmatine	Guan et al., 2017

## Prevention or induction of hepatotoxicity

Radioembolization-induced liver injury is an uncommon but relevant complication. Lycopene is an anti-oxidant substance in plants and micro-organisms. Its treatment significantly decreases radiotherapy-induced hepatic toxicity with oxidative stress in rats (Meydan et al., 2011). A perennial plant Ashwagandha (*Withania somnifera*), a perennial plant used in Ayurvedic medicine, shows hepatoprotective and anti-oxidant effects against radiation-induced hepatotoxicity in rats (Hosny Mansour and Farouk Hafez, 2012). *N. sativa* oil also alleviates irradiation-induced liver injury in rats (Radwan and Mohamed, 2018).

Chemotherapeutic agents commonly cause hepatic injury in cancer patients. Some Indian natural products have promise in relieving chemotherapy-induced hepatotoxicity (Metri et al., 2013). Recently, clinical trials show that milk thistle (main hepatoprotective ingredient silymarin) can effectively reduce hepatotoxicity in oncology patients (Frassova and Ruda-Kucerova, 2017). A edible mushroom mycelium product containing active hexose-correlated compound, can reduce hematotoxicity and hepatotoxicity, and improve the quality of life in patients with advanced cancer during chemotherapy (Ito et al., 2014).

In animals of chemotherapy-induced hepatotoxicity, Pine bark extract (Pycnogenol®), a standardized proprietary mixture of bioflavonoids extracted from the bark of *Pinus pinaster*, has protective effect due to its anti-oxidation (Ko et al., 2014). Pomegranate (*Punica granatum*) is mentioned in the Ebers papyrus of Egypt written in about 1550 BC. Its seed extract containing robust polyphenolic flavonoid can ameliorate cisplatin-induced liver damage in rabbits (Yildirim et al., 2013). Grape seed extract prevents against dexamethasone-induced liver histopathological change in rats (Hasona and Morsi, 2018). Ginsenoside Rg1, a main compound of Ginseng, effectively alleviates cisplatin-induced liver histological lesion in mice possibly by Nrf2 pathway (Gao et al., 2017). Turmeric and curcumin are known to have hepatoprotective action. They antagonize chemotherapy-induced hepatotoxicity (Mohamad et al., 2009; Waseem et al., 2014). Pre-treatment with a combination of curcumin and α-tocopherol regulates liver enzyme and lipid peroxidation biomarker, and alleviates liver histopathology change in rats, showing the protection against cisplatin-induced hepatotoxicity via abrogating oxidative stress (Palipoch et al., 2014). *Phyllanthus fraternus* has long been used in folk medicine to treat liver disturbance and painful disorder. *P. fraternus* attenuates cisplatin and cyclophosphamide-induced mitochondrial damage in rats with hepatotoxicity (Kumari and Setty, 2012). Thus, mitochondria may be considered as the target for the hepatoprotection (Waseem et al., 2017).

Of note, herbs are responsible for 24.2% of drug-induced liver injury cases (Li et al., 2007). Systematic review shows that a comprehensive list of herbs can cause a high risk of hepatotoxicity in Korea (Lee et al., 2015). *S. baicalensis* is reported to induce autoimmune hepatitis in mice through modulating immune response (Wang et al., 2014). Thus, natural products-induced liver injury is a hot topic. In a recent case report, Turmeric interaction with paclitaxel induces an acute toxic hepatitis in Caucasian male with lung cancer probably by altering pharmacokinetic of paclitaxel in association with a contaminated Chlorella supplement (Costa et al., 2018). These observations indicate that some natural products may evoke acute hepatotoxicity possibly via the interaction with chemotherapeutic agents. Although the hepatotoxicity of some natural products remains speculative, the associated liver injury address the need to clarify and investigate the potential harmful effects. The natural products in this part together with relevant characteristics of the respective studies are summarized in Table 3.

**Table 3:** Natural products in reducing chemotherapy and radiotherapy-induced hepatotoxicity.

**Name**	**Effect/Mechanism**	**Experimental setting/Model**	**Ingredients/Source**	**References**
Ashwagandha	Anti-oxidant	Radiation-induced hepatotoxicity in rats	Alkaloids, withanolides, and several sitoindosides	Hosny Mansour and Farouk Hafez, 2012
*N. sativa* oil	Reduction of oxidative stress, inflammatory, and fibrogenic markers	Irradiation-induced liver injury in rats	Rich in polyunsaturated fatty acids, such as omega-3 and omega-6 fatty acids, phytosterols, and several other substances including thymoquinone (up to 25% in volatile oil), carvacrol, t-anethole, sesquiterpenelongifolene and 4 terpinol	Radwan and Mohamed, 2018
Milk thistle	Anti-oxidation, Anti-inflammation, and anti-fibrosis	Patients	Silymarin	Frassova and Ruda-Kucerova, 2017
Active Hexose Correlated Compound	Anti-oxidantion, anti-inflammation, anti-tumor effect, anti-infectious effect and immunoenhancing activity	Patients	A culture extract of mycelium of *Lentinula edodes* (Basiomycetes family of fungi)	Ito et al., 2014
Pine bark extract	Anti-oxidation	Cisplatin-induced hepatotoxicity and oxidative stress in rats	Mixture of bioflavonoids	Ko et al., 2014
Pomegranate	Anti-oxidation	Cisplatin-induced liver damage in rabbits	Robust polyphenolic flavonoid	Yildirim et al., 2013
*P. fraternus*	Reduction of mitochondrial damage	Cisplatin and cyclophosphamide-induced hepatotoxicity and nephrotoxicity in rats	Alkaloids, flavonoids, lignans, phenols, and terpenes	Kumari and Setty, 2012
Grape seed extract	Reduction of oxidative stress, hyperlipidemia and hematological alterations	Dexamethasone-induced liver histopathological change in rats	A number of polyphenols, including procyanidins and proanthocyanidins	Hasona and Morsi, 2018
Ginsenoside Rg1	Regulation of Nrf2 signaling pathway	Cisplatin-induced hepatotoxicity in mice	A main compound of Ginseng	Gao et al., 2017
Turmeric	Induction of an acute toxic hepatitis by interacting with paclitaxel	Patients	An herbaceous perennial plant originated from Southeast Asia	Costa et al., 2018
*S. baicalensis*	Induction of autoimmune hepatitis	Mice	A principal constituents of Shosaikoto (or TJ-9)	Wang et al., 2014

## Capacity to relief nephrotoxicity

Nephrotoxicity is one of the most serious problem that hinders cisplatin-based chemotherapy (Mahmoodnia et al., 2017). In 2016, Shreesh Ojha et al. provide a review summarized plant-derived natural products for counteracting cisplatin-induced nephrotoxicity (Ojha et al., 2016). Of note, oxidative stress is significantly related to cisplatin-induced nephrotoxicity in head and neck cancer patients (Quintanilha et al., 2018). Lycopene has anti-oxidative effect. The results from a double-blind, randomized clinical trial show that lycopene is a useful adjuvant therapy, because its oral administration from 24 to 72 h after cisplatin administration can relief the complication of nephrotoxicity in cancer patients (Mahmoodnia et al., 2017). Hesperetin, a naturally-occurring bioflavonoid, protects against hydrogen peroxide-triggered oxidative damage via upregulation of Keap1-Nrf2/HO-1 pathway in ARPE-19 cells (Zhu et al., 2017). It normalizes renal function by attenuating cisplatin-induced oxidative stress, lipid peroxidation, inflammatory cytokine, and histopathological alteration in rats (Budhani et al., 2014). Fruits of *Withania coagulans* contain several bioactive compounds as curative agents for various clinical conditions. This fruit extract prevents against cisplatin-induced kidney damage, primarily through its free radical scavenging and anti-inflammatory activity (Sharma et al., 2017). Pine bark extract with potent anti-oxidant activity attenuates cisplatin-induced kidney injury of rats (Lee et al., 2017). Silybin (a main flavonoid of *S. marianum*) as a pharmacological activator of SIRT3, has capable of protection against cisplatin-induced tubular cell apoptosis and acute kidney injury by improving mitochondrial function (Li et al., 2017). *Origanum majorana* is commonly known as marjoram in traditional medicine, which possesses anti-oxidant, anti-microbial and anti-inflammatory activities (Leyva-Lopez et al., 2017). Its anti-proliferative effect is observed in human hepatocarcinoma cells (Fathy et al., 2016). Moreover, *O. majorana* and its polyphenol carnosol have anti-metastatic and antitumor effects in breast cancer cells (Al Dhaheri et al., 2013, 2014). Its ethanol extract significantly reduces cisplatin-induced nephrotoxicity in rats, possibly through enhancing free radical scavenging activity (Soliman et al., 2016).

Other chemotherapy agents such as adriamycin, 5-fluorouracil, methotrexate, ifosfamide and doxorubicin also cause nephrotoxicity. Traditional Chinese medicine Qi-Lu-Xiao-Bai decoction can inhibit connective tissue growth factor, fibronectin and α-smooth muscle actin expression, and improve glomerular sclerosis in adriamycin-induced nephropathy in rats (Su et al., 2015). Multi-glycoside of *Tripterygium wilfordii*, a Chinese herbal medicine, is clinically effective in improving glomerulosclerosis in chronic kidney disease. It effectively and safely relieves the prolonged glomerulosclerosis in adriamycin-induced nephropathy in rats, possibly via the reduction of extracellular matrix components and suppression of TGF-β1/Smad signaling (Wan et al., 2014). *N. sativa* and its constituent thymoquinone have potential renoprotective effect on chemotherapy-associated kidney complication, possibly via decreasing lipid peroxidation and increasing anti-oxidant enzyme activity (Cascella et al., 2017). Traditional herbal medicine *Thymus vulgaris* has anti-oxidant property (Heidari et al., 2018). Its anticancer effect is observed in human leukemia THP-1 cells and colorectal cancer cells (Ayesh et al., 2014; Al-Menhali et al., 2015). Naringenin, a natural flavanone isolated from *T. vulgaris*, has growth inhibitory and chemo-sensitization effects on human breast and colorectal cancer (Abaza et al., 2015). Naringenin can ameliorate daunorubicin-induced nephrotoxicity in rats by mitigating inflammation (Karuppagounder et al., 2015).

Kidney is a radiosensitive organ. *Acorus calamus* is a well-known plant in Asia traditional medicine for centuries. Its extract significantly increases major enzyme activity of the antioxidant defense system and decreases DNA strand breaks in liver and kidney of irradiated mice (Sandeep and Nair, 2012). The dried fruit of *Xylopia aethiopica* is used in folk medicines and widely consumed as a spice in Nigeria, with anti-plasmodial, anti-oxidant, hypotensive and diuretic effects (Tatsadjieu et al., 2003; Karioti et al., 2004). It increases the anti-oxidant defense system in liver and kidney of irradiated rats (Adaramoye et al., 2011). Quercetin also attenuates irradiation-induced kidney injury via its anti-oxidant activity (Ozyurt et al., 2014). Genistein, found in soybean products, has anti-oxidant and anti-inflammatory properties with low toxicity. Genistein is suggested be a chemotherapeutic agent against different types of cancer, mainly by changing apoptosis, cell cycle and angiogenesis and inhibiting metastasis (Spagnuolo et al., 2015). Melatonin is a potent free radical scavenger with anti-inflammation. The supplementation of genistein and melatonin prior to irradiation protects against nephrotoxicity in mice (Canyilmaz et al., 2016).

Nutritional supplement ingredients have attracted the attention for the ability to prevent and treat kidney injury. Honey and royal jelly are effective in reducing cisplatin-induced acute kidney injury in cancer patients (Osama et al., 2017). Ginger extract shows renoprotective effects on radiotherapy-induced renal histological and biochemical alteration in rats (Saberi et al., 2017). Purslane (*Portulaca oleracea*), widely distributed around the globe in traditional medicine, significantly attenuates lipid alteration, liver, and kidney function as well as oxidative stress in irradiated rats (Abd El-Azime et al., 2014). American ginseng berry extract also has nephroprotective effect against cisplatin-evoked nephrotoxicity in mice through ROS-mediated mitogen activated protein kinase (MAPK) and NF-κB signaling pathway (Ma et al., 2017). Curcumin and its analog difluorinated curcumin potentially reduce cisplatin-induced nephrotoxicity, thereby enhance the therapeutic window of cisplatin, the latter also decreases inflammatory factors NF-κB and COX-2, oxidative stress as well as multi-drug resistance markers (Sahin et al., 2014; Ugur et al., 2015). A Se-polysaccharide from Se-enriched *G. frondosa* (Se-GFP-22) significantly enhances the anti-oxidant and immunomodulatory activities in kidney of cyclophosphamide-treated mice (Li et al., 2018). Grape seed extract is found to reduce severe acute tubular necrosis and improve kidney function in cisplatin-treated rabbits, partially showing its protective effect against nephrotoxicity (Benzer et al., 2012). Recently, grape seed proanthocyanidin extract reduces thalidomide and carboplatin combination-induced renal damage in rats by reducing oxidative stress, inflammation, p53 change, and apoptosis (Yousef et al., 2018), as well as attenuates arsenic-induced renal inflammatory injury in mice by inhibiting NF-κB signaling activation and inflammatory cytokine production (Wang C. et al., 2017). Up to 2,500 mg of this extract administration for 4 weeks is found to be generally safe and well-tolerated in humans (Sano, 2017). These observations may offer a promising chance for clinically meaningful prevention of chemotherapy and radiotherapy-induced nephrotoxicity by natural products. The natural products in this part together with relevant characteristics of the respective studies are summarized in Table 4.

**Table 4:** Natural products in reducing chemotherapy and radiotherapy-induced nephrotoxicity.

**Name**	**Effect/Mechanism**	**Experimental setting/Model**	**Ingredients/Source**	**References**
Lycopene	Anti-oxidation	Patients	Available in tomatoes, tomato products, watermelons and grapefruit	Mahmoodnia et al., 2017
Hesperetin	Modulation of oxidative stress and renal inflammation	Cisplatin-induced nephrotoxicity in rats	A flavanone glycoside predominantly found in citrus fruits	Budhani et al., 2014
Fruits of *W. coagulans*	Free radical scavenging and anti-inflammation	Cisplatin-induced nephrotoxicity in rats	Withanolides, withaferin A, and coagulins	Sharma et al., 2017
Pine bark extract	Anti-oxidation	Cisplatin-induced kidney injury of rats	Mixture of bioflavonoids	Lee et al., 2017
Silibinin	Improvement of mitochondrial function through the regulation of SIRT3 expression	Male SV129 and SIRT3 knockout (KO) mice injection of cisplatin	A major active constituent of silymarin, a complex of flavonolignans extracted from milk thistle	Li et al., 2017
*O. majorana* ethanolic extracts	Free radical scavenging	Cisplatin-induced nephrotoxicity in rats	Phenolic terpenoids, flavonoids, tannins, hydroquinone, and phenolic glycosides	Soliman et al., 2016
Qi-Lu-Xiao-Bai decoction	Inhibition of connective tissue growth factor, fibronectin and α-smooth muscle actin expression, and improvement of glomerular sclerosis	Adriamycin-induced nephropathy in rats	Made from *Astragalus mongholicus bunge, Radix Rstragali preparata, Poria, Polygonum perfoliatum, Cornu cervi, Herba pyrolae* and *Cassia twig* Main chemical constituents: astragaloside, monotropein, pachymic acid and cinnamaldehyde	Su et al., 2015
Multi-glycoside of *T. wilfordii*	Reduction of extracellular matrix components and suppression of TGF-β1/Smad signaling	Adriamycin-induced nephropathy in rats	Multi-glycoside	Wan et al., 2014
*N. sativa*	Reduction of lipid peroxidation and increase of antioxidant enzyme activity	Experimental animal studies	Thymoquinone	Cascella et al., 2017
Naringenin	Mitigation of AT1R, ERK1/2-NFκB p65 mediated inflammation	Daunorubicin induced nephrotoxicity in rats	A natural flavanone purified from *T. vulgaris*	Karuppagounder et al., 2015
*A. calamus*	Increase of major enzyme activity of the antioxidant defense system and decrease of DNA strand breaks	Irradiated mice	Essential oils including (E)-asarone, gamma-asarone, (Z)-methyl isoeugenol and linalool	Sandeep and Nair, 2012
*X. aethiopica*	Increase of the antioxidant defense system	Irradiated rats	Rich in essential oils	Adaramoye et al., 2011
Quercetin	Anti-oxidation	Irradiated rats	A flavonoid found in fruits and vegetables	Ozyurt et al., 2014
Genistein and melatonin	Anti-oxidation	Radiation-induced nephrotoxicity in mice	Genistein is found in soybean products; Melatonin is a methoxyindole synthesized and secreted principally by the pineal gland	Canyilmaz et al., 2016
Honey and royal jelly	Anti-oxidation, hypoglycemic, anti-tumor, anti-inflammation, and antimicrobial effect	Patients	Glucose, fructose, acids, proteins, minerals, and polyphenols	Osama et al., 2017
Ginger extract	Anti-oxidation and anti-inflammation	Rats exposure to radiotherapy	Flovonoids, gingerol, shogaols, vitamin C, and dozens of polyphenolic compounds	Saberi et al., 2017
Purslane	Anti-oxidation and reduction of lipids alteration	Irradiated rats	Free oxalic acids, alkaloids (oleraceins A, B, C, D, and E), and omega-3 fatty acids	Abd El-Azime et al., 2014
American ginseng berry extract	Regulation of ROS-mediated mitogen activated protein kinase (MAPK) and NF-κB signaling pathway	Cisplatin-induced nephrotoxicity in mice	Ginsenosides, polysaccharides, volatile oil, and flavonoids	Ma et al., 2017
Curcumin	Increase of the NAMPT and SIRT protein levels	Cisplatin-treated rats	Bioactive constituent of *Curcuma longa* L.	Ugur et al., 2015
Difluorinated curcumin	Reduction of inflammatory factors NF-κB and COX-2, oxidative stress as well as multi-drug resistance markers organic cation transporters	Cisplatin-induced nephrotoxicity in rats	Curcumin analog	Sahin et al., 2014
Se-enriched G. frondosa (Se-GFP-22)	Anti-oxidation	Cyclophosphamide-treated mice	Se-polysaccharide	Li et al., 2018
Grape seed proanthocyanidin extract	Reduction of renal damage, oxidative stress, inflammation, tumor suppressor protein p53 change, as well as renal cell apoptosis; inhibition of NF-κB signaling pathway and inflammatory cytokine production	Thalidomide and carboplatin-treated rats	Proanthocyanidin	Sano, 2017; Wang C. et al., 2017; Yousef et al., 2018

## Improvement of hematopoietic system injury, particularly important for bone marrow hematopoietic microenvironment

Myelosuppression with leukocytopenia, erythrocytopenia, and thrombocytopenia, are serious and common side effects during cancer treatment. A systematic review and meta-analysis of randomized controlled trials show that Chinese herbal medicine as an adjuvant can alleviate chemotherapy or radiotherapy-induced myelosuppression, and maintain therapeutic dose and treatment cycle by reducing grade III-IV toxicity, (Hou et al., 2017). A Chinese herbal medicine Shuang-Huang-Sheng-Bai granule approved by the Food and Drug Administration of China, can effectively treat cancer. Recently, this granule is observed to elevate white blood cell count, and reduce the incidence of chemotherapy-caused myelosuppression in cancer patients compared with control group taken with Leucogon tablet, demonstrating its protection against bone marrow suppression and alleviation of clinical symptoms (Wang L. F. et al., 2017).

Cyclophosphamide is of great interest in the clinic due to its relatively high oncotoxic specificity. Sheng-Mai injection is derived from Sheng-Mai-San, a well-known traditional Chinese herbal prescription. This injection has therapeutic potential in reducing chemotherapy-induced adverse effects and improving life quality in patients with non-small cell lung cancer. Recently, a meta-analysis shows that combination of Sheng-Mai injection with chemotherapy significantly reduces grade 3/4 myelosuppression compared with the chemotherapy alone in cancer patients (Duan et al., 2018). Chinese Ginseng is well-known to strengthen the body resistance to eliminate pathogenic factors, and reduce side effects of chemotherapy drugs. Panaxadiol saponins, a biologically active fraction derived from Ginseng, possess hematopoietic growth factor-like activity that promotes proliferation and differentiation of HPCs in cyclophosphamide-induced myelosuppressive mice, probably by regulating MAPK/ERK kinase (MEK) and extracellular signal-regulated kinase (ERK) protein kinases, C-kit, and GATA-1 transcription factors (Sun X. et al., 2018). Ginsenoside Rg3 is used as a potent anticancer agent to induce apoptosis, inhibit proliferation, metastasis and angiogenesis, as well as promote immunity during conventional cancer therapy (Sun et al., 2017). A multicenter, large-sample, randomized clinical trial shows that ginsenoside Rg3 improves the median survival time and reduces myelosuppression in advanced non-small cell lung cancer patients during the standard first-line chemotherapy (Zhang et al., 2018). Dang-Gui-Bu-Xue decoction, a classical formula of traditional Chinese medicine, has an impact on promoting hematopoiesis, and prevents myelosuppression in breast cancer patients treated with adjuvant chemotherapy. However, a phase II randomized prospective controlled clinical study is conducted from December 2013 to February 2015, and shows that this decoction fails to prevent myelosuppression in breast cancer patients treated with adjuvant chemotherapy (Hong et al., 2017). Further studies are warranted to validate the efficacy of Dang-Gui-Bu-Xue decoction in selected patients.

Infection and inflammation are observed in chemotherapy and radiotherapy patients with myelosuppression. A traditional medicinal formula Dang-Gui-Si-Ni decoction has been clinically used for infectious diseases that are complicated with hemodynamic instability (Yao et al., 2014). This decoction significantly elevates the level of bone marrow hematopoietic stem progenitor cells in myelosuppression model of mice (Chen et al., 2015). *Herba Epimedii*, one of most popular Chinese herbs, is used for the treatment of osteoporosis and inflammation. *H. Epimedii* and its main constituent icariin can improve immune function after cyclophosphamide-induced myelosuppression (Zhao et al., 2010). Icaritin, hydrolyzed by icariin, prevents cyclophosphamide-induced myelosuppression in mice by improving bone marrow hematopoietic microenvironment, promoting the proliferation and differentiation of HSCs, inhibiting the apoptosis of HSCs and stimulating granulocyte colony-stimulating factor and thyroperoxidase (Sun C. et al., 2018). A herbal medicine *Radix Sanguisorbae* used to treat diarrhea, enteritis, duodenal ulcers, and internal hemorrhage, is clinically effective against myelosuppression induced by chemotherapy and/or radiotherapy (Seo et al., 2016). Its main ingredients saponins show hematopoietic effect mediated by focal adhesion kinase (FAK) and ERK1/2 activation as well as cytokine inhibition in the bone marrow (Chen X. et al., 2017). Paeoniflorin and albiflorin, two active constituents identified from the root of *P. lactiflora*, can increase the white blood cell counts, reverse the atrophy of thymus, and suppress cyclophosphamide and radiotherapy-induced myelosuppression in animals, showing hematopoietic effect (Zhu Y. et al., 2016; Zhu Y.-L. et al., 2016).

Anti-oxidant natural products can prevent excessive ROS produced by chemotherapy and radiotherapy from killing white blood cells and inhibiting bone marrow suppression. Chinese medicine Yi-Qi-Yang-Yin formula can ameliorate hematopoietic system injury by reducing intracellular ROS levels in hematopoietic cells of mice after total body irradiation (Zhang et al., 2017). San-Yang-Xue-Dai mixture is a natural medicine originating from an ancient prescription of the Dai nationality in Southwest China. It attenuates doxorubicin-induced myelosuppression by inhibiting ROS-mediated apoptosis (Chen T. et al., 2017). Coriander (*Coriandrum sativum*) is an annual herb used as a flavoring agent and traditional remedy. Its extract can scavenge ROS and up-regulate endogenous cellular antioxidant system (Velaga et al., 2014; Zielniok et al., 2016). Recently, rutin-enriched coriander extract is found to ameliorate ionizing radiation-induced myelosuppression with improvement of the proliferation and differentiation ability of hematopoietic stem and progenitor cells in mice, probably by inhibiting apoptosis and DNA damage attributed to its scavenging ROS and activating antioxidant enzyme ability (Han et al., 2017a). Theaflavin, one of the tea pigments from black tea, prevents the progression of inflammatory disorder, cancer, bacterial and viral infection, and ameliorates ionizing radiation-induced HSC injury in mice by regulating Nrf2 pathway to reduce oxidative stress (Han et al., 2017b). Astaxanthin, predominantly found in marine organisms, can improve radiation-induced skewed differentiation of peripheral blood cells and accelerate hematopoietic self-renewal and regeneration in mice. This radio-protective effect is probably mediated by scavenging of ROS, activation of Nrf2 and downstream anti-oxidative proteins (Xue et al., 2017). The natural products in this part together with relevant characteristics of the respective studies are summarized in Table 5.

**Table 5:** Natural products in reducing chemotherapy and radiotherapy-induced hematopoietic system injury.

**Name**	**Effect/Mechanism**	**Experimental setting/Model**	**Ingredients/Source**	**References**
Shuang-Huang-Sheng-Bai granule	Elevation of white blood cells, promotion of the proliferation and differentiation of hematopoietic stem/progenitor cells, and the growth of bone marrow hematopoietic cells, inhibition of tumor cell growth and some immunomodulatory effects	Patients	Made from *Astragalus membranaceus, Rhizoma polygonati, Rhizoma drynariae, Fructus ligustri lucidi, Radix trichosanthes*, and *Herba epimedii* Main chemical constituents: astragaloside, polysaccharides, naringin, specnuezhenide, and icariin	Wang L. F. et al., 2017
Sheng-Mai injection	Improvement in quality of life, increase of the cellular immunity	Patients	Made from *Radix ginseng, Radix ophiopogonis*, and *Fructus schisandrae chinensis* Main chemical constituents: *g*insenoside Rg1, ginsenoside Re, ginsenoside Rb1, ophiopogonin D, ophiopogonin D', ophiogonanone A and ophiogonanone B	Duan et al., 2018
Panaxadiol saponins	Regulation of MEK and ERK protein kinases, C-kit, and GATA-1 transcription factors	Cyclophosphamide- treated mice	Derived from Ginseng	Sun X. et al., 2018
Ginsenoside Rg3	Induction of apoptosis, inhibition of proliferation, metastasis, and angiogenesis, promotion of immunity	Patients	A main compound of Ginseng	Zhang et al., 2018
Dang-Gui-Bu-Xue decoction	No prevention of myelosuppression in breast cancer patients	Patients	Made from *Radix astragali* and *Radix Angelicae sinensis* Main chemical constituents: calycosin-7-glucoside, ononin, calycosin, formononetin, Z-ligustilide, astragaloside IV, astragaloside II and astragaloside I	Hong et al., 2017
Dang-Gui-Si-Ni decoction	Upregulation of thrombopoietin expression	Myelosuppression model of mice	Made from *Angelica sinensis, Cinnamomi cassia, Paeonia lactiiflora, Tetrapanax papyriferus, Asarum heterotropoides, Glycyrrhiza uralensis* and *Ziziphus jujuba* Main chemical constituents: ferulic acid, paeoniflorin, cinnamic acid, and glycyrrhizic acid	Chen et al., 2015
Icaritin	Promoting the proliferation and differentiation of hematopoietic stem cells, inhibition of apoptosis and stimulating the expression of granulocyte colony-stimulating factor and thyroperoxidase	Cyclophosphamide-induced myelosuppression in mice	Hydrolyzed by icariin	Sun C. et al., 2018
Saponins	Activation of focal adhesion kinase (FAK) and Erk1/2, inhibition of the cytokine expression	Myelosuppressive mice	A main ingredients of *Radix Sanguisorbae*	Chen X. et al., 2017
Paeoniflorin and albiflorin	Increase of the white blood cell counts, attenuation of the atrophy of thymus	Cyclophosphamide and radiotherapy-induced myelosuppression in animals	Active constituents derived from the root of *P. lactiflora*	Zhu Y. et al., 2016; Zhu Y.-L. et al., 2016
Yi-Qi-Yang-Yin formula	Anti-oxidation	Mice after total body irradiation	Made from *Astragalus* root, Ginseng, glossy privet fruit, *Eclipta alba*, Chinese *Angelica*, Bighead *Atractylodes* rhizome, *Wolfiporia extensa*, and Radix *Glycyrrhizae* Preparata Main chemical constituents: astragaloside, ginsenoside, specnuezhenide, ferulic acid, and glycyrrhizic acid	Zhang et al., 2017
San-Yang-Xue-Dai mixture	Inhibition of ROS-mediated apoptosis	Doxorubicin-treated mice	Made from *Sanguis draconis, Radix et Rhizoma Notoginseng, Radix et Rhizoma Glycyrrhizae* and *Radix Angelicae Sinensis* Main chemical constituents: bornyl acetate, dracorhodin, ferulic acid, glycyrrhizic acid, 6-gingerol, ginsenoside Rg1, ginsenoside Rb1 and notoginsenoside R1	Chen T. et al., 2017
Rutin-enriched coriander extract	Inhibition of ROS-mediated apoptosis and DNA damage	Ionizing radiation-induced hematopoietic injury of mice	Phenolic acids (caffeic acid, protocatechinic acid, and gentisic acid), glycitin, and pyrogallol	Han et al., 2017a
Theaflavin	Anti-oxidation via the Nrf2 pathway	Ionizing radiation-induced HSC injury in mice	Tea pigments from black tea	Han et al., 2017b
Astaxanthin	Anti-oxidation, activation of Nrf2 and anti-oxidative proteins	Radiation-induced mice	Found in marine organisms	Xue et al., 2017

## Attenuation of cardiotoxicity mainly by balancing energy metabolism and antioxidant system

Energy metabolism imbalance and oxidative stress mediate chemotherapeutic agents-induced cardiotoxicity. Doxorubicin has cumulative and dose-related cardiotoxicity. In 2017, Sahebkar et al. review the attenuation of doxorubicin-induced cardiotoxicity by plant extracts and phytochemicals (Hosseini and Sahebkar, 2017). In 2018, Yu et al. also review the protective effects of natural products on doxorubicin-caused cardiotoxicity without affecting its anticancer efficacy (Yu et al., 2018b). A Chinese medicine Dan-Hong injection is demonstrated to alleviate ischemic myocardial injury and improve heart function. Recently, this injection is reported to restore doxorubicin-induced cardiotoxicity in H9c2 cells by improving energy metabolism and reducing oxidative stress (Yi et al., 2018). San-Yang-Xue-Dai mixture alleviates doxorubicin-induced cardiotoxicity and apoptosis by inhibiting p53 and MAPK signaling activation (Chen et al., 2018). Fermented *Cordyceps sinensis* is reported to attenuate doxorubicin-induced cardiotoxicity by inhibiting myocardial hypertrophy and myocardial damage, regulating systolic function, and antioxidant enzyme system and improving cardiac energy metabolism (Wu et al., 2018). Chinese herb *Salvia miltiorrhiza* is used as an empiric treatment for cardiovascular disorders (Zhou et al., 2012). Its main compound diethyl blechnic inhibits doxorubicin-induced apoptosis by inhibiting ROS in H9c2 cells and primary rat cardiomyocytes (Yu et al., 2018a). Curcumin also protects the myocardium against doxorubicin-induced cardiotoxicity in mouse hearts and primary cardiomyocytes, probably via upregulating 14-3-3γ expression (He et al., 2018). Natural steroid saponin dioscin found abundantly in legumes and yams, alleviates doxorubicin-induced cardiotoxicity by regulating miR-140-5p-mediated myocardial oxidative stress (Zhao et al., 2018).

Anthracycline also causes cardiotoxicity in cancer patients. Modified Zhi-Gan-Cao-Tang relieves anthracycline-induced congestive heart failure in an 18-year-old adolescent male (Wu et al., 2015). *Platycodon grandiflorum* is used in traditional Chinese medicine for centuries to treat cardiovascular disease. It can nourish *Qi* and relieve symptoms such as palpitations, shortness of breath, and chest pain. A randomized controlled trial reveals that *P. grandiflorum* has cardioprotective effect in early breast cancer patients undergoing anthracycline-based chemotherapy (Hao et al., 2017). Flaxseed oil (containing α-linolenic acid) reduces arsenic-induced cardiac toxicity in rats (Varghese et al., 2017). Parsley oil with anti-oxidant, anti-inflammatory and anti-apoptotic actions is reported to ameliorate cisplatin-induced hepatic and cardiac injury in rats (Abdellatief et al., 2017). Blueberry anthocyanins-enriched extract with anti-inflammatory and anti-oxidant activities attenuates cyclophosphamide-induced cardiotoxicity of rats (Liu et al., 2015). *Rubia cordifolia* is a valuable medicinal herb in the Ayurvedic system. Its extract can protect cyclophosphamide-induced cardiac tissue injury of rats by modulating anti-oxidant markers (Chandrashekar et al., 2018).

Radiotherapy-driven heart injury remains a major issue for cancer survivors. A traditional Chinese medicine Sheng-Mai-San is used to improve the syndrome of *Qi* and *Yin* deficiency, and has the ability to treat patients with cardiac diseases, fatigue and cancer. It also enhances heart function and improves the quality of life in cancer patients undergoing chemotherapy or radiotherapy (Lo et al., 2012). Black grape juice protects against whole body γ-irradiation-induced heart toxicity of rats with the alteration of metabolites from lipid peroxidation and lactate dehydrogenase (de Freitas et al., 2013). A natural polyphenol zingerone due to its anti-oxidative and anti-inflammatory properties can prevent against cisplatin- or γ-radiation-induced cardiotoxicity in rats by decreasing caspase-3 expression and the prominent nuclear DNA fragmentation as well as increasing mitochondrial complexes' activities (Soliman et al., 2018). A citrus flavanoglycone hesperidin also restores whole body γ-irradiation-induced cardiocellular damage and oxidative stress in rats (Pradeep et al., 2012). These observations suggest that natural products may be the effective agents used as an adjunct/dietary supplement for the cancer patients receiving chemotherapy and radiotherapy. The natural products in this part together with relevant characteristics of the respective studies are summarized in Table 6.

**Table 6:** Natural products in reducing chemotherapy and radiotherapy-induced cardiotoxicity.

**Name**	**Effect/Mechanism**	**Experimental setting/Model**	**Ingredients/Source**	**References**
Sheng-Mai-San	Improvement of the syndrome of qi and yin deficiency, heart function and the quality of life of cancer patients	Patients	Made from *Ginseng radis, Liriope spicata*, and *Schizandrae fructus* Main chemical constituents*:* ginsenoside Rg, ginsenoside Re and schisandrin	Lo et al., 2012
Dan-Hong injection	Improvement of energy metabolism and reduction of oxidative stress	Doxorubicin-induced cardiotoxicity in H9c2 cells	Made from *Radix Salviae miltiorrhizae* and *Flos Carthami tinctorii* Main chemical constituents: catechol, tanshinone, salvianic aid A, Carthamin and carthamin yellow	Yi et al., 2018
San-Yang-Xue-Dai mixture	Inhibition of ROS-mediated p53 and MAPK signal pathways	Doxorubicin-induced cardiotoxicity in mice	Made from *Daemonorops draco, Panax notoginseng, Scoparia dulcis, Aralia cordata, Alpinia ofcinarum, Dioscorea opposita, Wolfporia extensa*,and *Amomum villosum*	Chen et al., 2018
Fermented *C. sinensis*	Inhibition of myocardial hypertrophy and myocardial damage, Improvement of systolic function, the antioxidant enzyme system, and cardiac energy metabolism, upregulation of the cAMP and AMPK signaling pathways	Doxorubicin-induced cardiotoxicity in rats	Protein, carbohydrate, fat, ash, cordycepin, H2O, amino acid, and adenosine	Wu et al., 2018
Diethyl blechnic	Anti-oxidation	Doxorubicin-induced apoptosis c and primary rat cardiomyocytes	A main compound isolated from *S. miltiorrhiza*	Yu et al., 2018a
Curcumin	Upregulation of 14-3-3γ expression	Doxorubicin-induced cardiotoxicity in mice and primary cardiomyocytes	Bioactive constituent of Curcuma longa L.	He et al., 2018
Saponin dioscin	Regulation of miR-140-5p-mediated myocardial oxidative stress	Doxorubicin-induced cardiotoxicity in H9c2 cells and rats	Found abundantly in legumes and yams	Zhao et al., 2018
Modified Zhi-Gan-Cao-Tang	Nourish heart yin and yang, anti-oxidation and inhibition of Na^+^/K^+^-ATPase	Anthracycline-induced congestive heart failure in an 18-year-old adolescent male	*Radix Glycyrrhizae, Radix Ginseng, Fructus Jujubae, Radix Rehmanniae, Radix Ophiopogonis, Colla Corii Asini, Fructus Cannalis, Ramulus Cinnamomi, Rhizoma Zingiberis Recens, Carapax Trionycis, Plastrum Testudinis, Concha Ostreae*, and *Radix Paeoniae alba*	Wu et al., 2015
*P. grandiflorum*	Anti-oxidation	Early breast cancer receiving anthracycline-based chemotherapy	Triterpenoid saponin, carbohydrates, and fibers	Hao et al., 2017
Flaxseed oil	Maintenance of the proper balance between pro-oxidant/antioxidant defense systems	Arsenic-induced cardiac toxicity in rats	Polyunsaturated fatty acid alpha-linolenic acid	Varghese et al., 2017
Parsley oil	Anti-oxidation and anti-inflammation and anti-apoptotic	Cisplatin-induced hepatic and cardiac injuries in rats	Phenolic compounds, particularly flavonoids (e.g., apigenin, apiin, and 600-acetylapiin), coumarins, furocoumarins, and essential oil components (mainly myristicin and apiol)	Abdellatief et al., 2017
Blueberry anthocyanins-enriched extracts	Anti-oxidation and anti-inflammation	Cyclophosphamide-induced cardiac injury in rats	3-glycosidic derivatives of cyanidin, delphinidin, malvidin, petunidin, and peonidin	Liu et al., 2015
*R. cordifolia*	Anti-oxidation	Cyclophosphamide-induced cardiac injury in rats	Alkaloids, flavonoids, saponins, and anthraquinones	Chandrashekar et al., 2018
Black grape juice	Anti-oxidation	Whole body γ-irradiation-induced heart toxicity of rats	Phenolics, flavonoids, tannin, gallic acid, catechin, resveratrol, caffeic acid, ellagic acid, quercetin, kaempferol	de Freitas et al., 2013
Sheng-Mai Zingerone	Decrease of caspase-3 gene expression and the prominent nuclear DNA fragmentation as well as increase of mitochondrial complexes' activities	Cisplatin- or γ-radiation-induced cardiotoxicity in rats	A active components of ginger	Soliman et al., 2018
Hesperidin	Inhibition of cellular damage and oxidative stress	γ-radiation-induced tissue damage in Sprague-Dawley rats	Isolated from the ordinary orange Citrus aurantium and other species of the genus Citrus	Pradeep et al., 2012

## Need to clarify the prevention of neurotoxicity

Neurotoxicity is a frequent adverse effect of cancer chemotherapy and radiotherapy, and causes excruciating pain to cancer patients. A network meta-analysis shows that Ai-Di, Shen-Qi-Fu-Zheng, and Matrine injections approved by the Food and Drug Administration of China, improve the overall response rate and quality of life, and reduce the incidence of peripheral neurotoxicity (III-IV) for advanced colorectal cancer treated with oxaliplatin, 5-fluorouracil, and leucovorin (Ge et al., 2016). Traditional Japanese medicine Goshajinkigan (TJ107) is used to alleviate neuropathy and general pain. Shakuyakukanzoto (TJ68) effectively treats muscle cramps and crampy pain. A multicenter retrospective study shows that TJ107 and TJ68 reduce neurotoxicity without negatively affecting tumor response in patients with colorectal cancer who undergo 5-fluorouracil/folinic acid plus oxaliplatin therapy (Hosokawa et al., 2012). A systematic review and meta-analysis shows that *Radix Astragali* intervention may be beneficial in reducing oxaliplatin-induced peripheral neuropathy (Deng et al., 2016a). Recently, its hydroalcoholic extract (containing astragalosides) is reported to relieve pain and promote the rescue mechanisms for the protection of nervous tissue in oxaliplatin-induced neuropathy of rats (Mannelli et al., 2017) as well as prevents against oxaliplatin-induced lipid peroxidation and DNA oxidation in astrocytes (Mannelli et al., 2015), further demonstrating the anti-neuropathic profile of *Radix Astragali*.

Currently, chemotherapy and radiotherapy-induced peripheral neuropathy is focused on the treatment with anti-convulsants, anti-depressants, opioids, and non-opioid analgesics. A traditional Chinese medicine Wen-Luo-Tong, has been used to alleviate oxaliplatin-induced neuropathic pain for many years. Recently, Wen-Luo-Tong is found to prevent glial activation and nociceptive sensitization in a rat model of oxaliplatin-induced neuropathic pain (Deng et al., 2016b). Its ingredients including hydroxysafflor yellow A, icariin, epimedin B, and 4-dihydroxybenzoic acid increase the viability of Schwann cells injured by oxaliplatin. Microemulsion formulation containing these ingredients also decreases oxaliplatin-induced mechanical hyperalgesia responses in rat model (Lin et al., 2017). Liu-Jun-Zi-Tang is a traditional herbal medicine widely used in East Asia and clinically applied to treat functional dyspepsia and depression. Recently, it is reported to attenuate cisplatin-induced thermal hyperalgesia in mice and apoptosis in human neuroblastoma SH-SY5Y cells, showing its prevention of cisplatin-induced neurotoxicity, possibly through anti-oxidation and mitochondrial function regulation (Chiou et al., 2018). A Mexican medicinal plant *Tithonia tubaeformis* is used for the treatment of rheumatism and stomachache. Its hydromethanolic extract is effective in attenuating vincristine-induced allodynia and thermal hyperalgesia in mice, relieving chemotherapy-induced peripheral neuropathy (Nawaz et al., 2018). *Hypericum perforatum* (St. John's Wort) is used for centuries as a natural remedy for the treatment of a variety of disorders including depression (Asgary et al., 2012; Abtahi Froushani et al., 2015). It can reduce oxaliplatin-induced caspase-3 activity in rat astrocytes, but its alone produces a cytotoxic effect and fails to reduce the cytotoxicity of oxaliplatin in HT-29 cancer cells (Cinci et al., 2017). Its main constituent hypericin is a potent inhibitor of glioma growth *in vitro*. In patients with documented recurrent or progressive malignant gliomas who have received standard radiation therapy with or without chemotherapy, oral hypericin is well-tolerated (Couldwell et al., 2011), suggesting that *H. perforatum* is used as a therapeutic strategy for counteracting chemotherapy and radiotherapy-induced neuropathy. Natural bicyclic sesquiterpenes, β-caryophyllene and β-caryophyllene oxide are found in a large number of plants worldwide. They possess neuropharmacological effects as chemo-sensitizing agents for doxorubicin chemotherapy and re-sensitize cancer-resistant cells (Di Giacomo et al., 2017). A therapeutic plant Lithospermi radix (the root of *Lithospermum erythrorhizon*) is used to treat septic shock, eczema, and burns. Its water extract restores oxaliplatin-induced neurotoxicity in nerve growth factor-stimulated neurite outgrowth in PC12 cells and animals with enhanced nociceptive sensitivity to mechanical stimuli along with spinal activation of microglias and astrocytes as well as loss of intraepidermal nerve fibers of footpads (Cho et al., 2016). β-Caryophyllene also effectively attenuates paclitaxel-induced peripheral neuropathy in mice, possibly through CB_2_-activation in the central nervous system and posterior inhibition of p38 MAPK/NF-κB activation (Segat et al., 2017). Curcumin also has neuroprotective action. In 2017, Rezaee et al. provide a summary of the studies done to show the protective effects of curcumin against cisplatin-induced neurotoxicity, nephrotoxicity and ototoxicity (Rezaee et al., 2017). Its natural derivative demethoxycurcumin exhibits neuroprotective effect in rotenone-induced neurotoxicity in SH-SY5Y neuroblastoma cells (Ramkumar et al., 2017). 5, 7-Dihydroxyflavone (Chrysin), a natural plant flavonoid, has neuroprotective effect against γ-irradiation induced-neurotoxicity in rats (Mansour et al., 2017). Shikonin, a natural naphthoquinone compound, is one of the main chemicals isolated from *Lithospermi radix*. Shikonin and its derivatives suppress the epidermal growth factor receptor signaling and synergistically kill glioblastoma cells in combination with erlotinib, possibly being a potential strategy to overcome drug resistance to erlotinib (Zhao et al., 2015). Oxidative damage contributes to cisplatin-induced neurotoxicity. As an apoptotic inhibitor, natural flavonoid compound cyanidin effectively restores cisplatin-induced neurotoxicity through inhibition of ROS-mediated DNA damage and apoptosis (Li et al., 2015).

Dietary supplement OPERA® containing α-lipoic acid, Boswellia Serrata, methylsulfonylmethane and bromelain, is able to improve chemotherapy-induced peripheral neuropathy symptoms in a prospective series of patients treated with neurotoxic chemotherapy, without significant toxicity or interaction (Desideri et al., 2017). A systematic review of preclinical studies demonstrate that grape seed extract can treat chemotherapy and radiotherapy-induced neurotoxicity (Olaku et al., 2015). Clinical trial demonstrates that green tea consumption with neuroprotective effect has anticancer action via regulation of intra-tumoural lymph-angiogenesis and COX-2 expression (Najaf Najafi et al., 2018). These diet supplements may be effective in the prevention or treatment of chemotherapy and radiotherapy-induced neurotoxicity.

However, in 2017, Schloss et al. provide review that there is no solid beneficial evidence for herb medicine which is recommended for the prevention or treatment of chemotherapy-induced peripheral neuropathy (Schloss et al., 2017). In fact, in 2015, Oki et al. conduct a placebo-controlled, double-blind, randomized phase III study and observe no effects of Goshajinkigan on oxaliplatin-associated peripheral neuropathy in patients with colorectal cancer (Oki et al., 2015). Consistently, in 2017, Kuriyama et al. conduct a systematic review and meta-analysis, and further demonstrate that Goshajinkigan is impossible to prevent peripheral neuropathy in patients receiving neurotoxic chemotherapy. Next, Hoshino et al. also conduct a systematic review and show that Goshajinkigan tends to prevent persistence but not severity of chemotherapy-induced peripheral neuropathy (Hoshino et al., 2018). Given the low quality and insufficient amount of the evidence, use of Goshajinkigan as standard of care is not currently recommended (Kuriyama and Endo, 2018). On the other hand, although phytochemicals, medicinal herbs, and their formulas may be considered for prophylaxis of chemotherapy-induced peripheral neuropathy, the curative usability as well as the reciprocal effect with other drug should be examined in well-designed clinical trials (Lee and Kim, 2016). The natural products in this part together with relevant characteristics of the respective studies are summarized in Table 7.

**Table 7:** Natural products in reducing chemotherapy and radiotherapy-induced neurotoxicity.

**Name**	**Effect/Mechanism**	**Experimental setting/Model**	**Ingredients/Source**	**References**
Ai-Di injection	Improvement of overall response rate and quality of life, reduction of the incidence of peripheral neurotoxicity (III-IV)	Patients	made from the extracts of *Mylabris phalerata, Radix astragalus, Radix ginseng*, and *Acanthopanax senticosus*	Ge et al., 2016
Shen-Qi-Fu-Zheng injection	Improvement of overall response rate and quality of life, reduction of the incidence of peripheral neurotoxicity (III-IV)	Patients	Made from the extracts of *Radix Astragali* and *Radix Codonopsis*	Ge et al., 2016
Matrine injection	Improvement of overall response rate and quality of life, reduction of the incidence of peripheral neurotoxicity (III-IV)	Patients	Major component of the traditional Chinese herb Sophora flavescens	Ge et al., 2016
TJ107 and TJ68	TJ107 has antinociceptive effects caused by increased nitric oxide production and induction of dynorphin release in the spinal cord; the antinociceptive effects of TJ68 have been attributed to the activation of spinal-descending noradrenergic neurons	Patients	TJ107 consists of *Rehmanniae radix, Achyranthis radix, Corni fructus, Moutan cortex, Alismatics rhizome, Dioscoreae rhizome, Plantaginis semen, Hoelen*, processed *Aconiti tuber*, and *Cinnamomi cortex*, which includes magnesium stearate, lactose, and fructose fatty acid esters as diluents TJ68 is composed of *Paeoniae Radix and Glycyrrhizae Radix* Main chemical constituents: albiflorin, paeoniflorin, glycycoumarin, isoliquiritigenin, glycyrrhetic acid, and glycyrrhetic acid-3-O-monoglucuronide	Hosokawa et al., 2012
*Radix Astragali*	Relieve of pain and promote of the rescue mechanisms	Oxaliplatin-induced neuropathy of rats	Astragalosides	Mannelli et al., 2017
Wen-Luo-Tong	Unknown	Oxaliplatin-treated rats and Schwann cells	Made from Epimedium herb, Geranium wilfordii, Cassia twig and Carthamus tinctorius Main chemical constituents: hydroxysafflor yellow A, icariin, epimedin B and 4-dihydroxybenzoic acid	Lin et al., 2017
Liu-Jun-Zi-Tang	Anti-oxidation and mitochondrial function regulation	Cisplatin treated mice and human neuroblastoma SH-SY5Y cells	Made from *Ginseng, Atractylodes, Poria cocos, Glycyrrhizae, Pericarpium Citri Reticulatae*, and *Pinellia ternata* Main chemical constituents: succinic acid, hesperidin, ginsenoside Rb1, glycyrrhizic acid I, 2-atractylenolide and pachymic acid	Chiou et al., 2018
*T. tubaeformis*	Anti-nociception	Vincristine-treated mice	Tagitinin A-C and F, diversifol and tithonine chromene, and flavone derivatives	Nawaz et al., 2018
*H. perforatum*	Glioma growth inhibition	Patients	Hypericin, pseudohypericin, flavonoids, oligomeric procyanidins, and hyperforin	Couldwell et al., 2011
β-caryophyllene	CB_2_-activation and inhibition of p38 MAPK/NF-κB activation	Paclitaxel-treated mice	Found in various plants	Segat et al., 2017
Lithospermi radix	Anti-inflammation	PC12 cells and animals induced by oxaliplatin	Furylhydroquinone derivatives and shikonin	Cho et al., 2016
Curcumin	Reduction of lipid peroxidation, maintenance of the balance of catalase, glutathione peroxidase and superoxide dismutase, and anti-oxidation	Rats	Bioactive constituent of Curcuma longa L.	Rezaee et al., 2017
Demethoxycurcumin	Suppression of apoptosis by regulating pro and anti-apoptotic indices and attenuating oxidative stress and mitochondrial dysfunction	Rotenone-induced neurotoxicity in SH-SY5Y neuroblastoma cells	Curcumin analog	Ramkumar et al., 2017
5, 7-Dihydroxyflavone (Chrysin)	Anti-lipid peroxidative, anti-amyloidogenic, and anti-apoptotic effects	Irradiation induced-neurotoxicity in the brain of rats	A flavonoid content extracted from propolis, honey, and plants	Mansour et al., 2017
Shikonin	Suppression of the epidermal growth factor receptor signaling and killing of glioblastoma cells	U87MG cells	Main chemicals isolated from *Lithospermi radix*	Zhao et al., 2015
Cyanidin	Inhibition of ROS-mediated DNA damage and apoptosis	PC12 cells	A flavonoid derived from cherry	Li et al., 2015
Dietary supplement OPERA^®;^	Anti-oxidation, anti-inflammation, anti-atherosclerotic, and anti-thrombotic effect	Patients	α-lipoic acid, boswellia serrata, methylsulfonylmethane, and bromelain	Desideri et al., 2017
Grape seed proanthocyanidins	Anti-oxidation	Mice, rats, and cells	Rich in polyphenols of which about 60% to 70% is found in grape seeds as dimers, trimers, and other oligomers of flavan-3-ols, known commonly as proanthocyanidins	Olaku et al., 2015
Green tea	Regulation of intra-tumoural lymph-angiogenesis and expression of COX-2	Patients	Catechins	Najaf Najafi et al., 2018
Goshajinkigan (TJ107)	No effects of Goshajinkigan on oxaliplatin-associated peripheral neuropathy in patients with colorectal cancer	Patients	*Rehmanniae radix, Achyranthis radix, Corni fructus, Moutan cortex, Alismatics rhizome, Dioscoreae rhizome, Plantaginis semen, Hoelen*, processed *Aconiti tuber*, and *Cinnamomi cortex*	Oki et al., 2015; Hoshino et al., 2018; Kuriyama and Endo, 2018

## Limitations

Recent efforts to prevent chemotherapy and radiotherapy-induced side effects have used natural products with biochemical and pharmacological properties. However, there are multiple limitations. These include:

- Lack of high quality trials using multiple measures to assess the various aspects of chemotherapy and radiotherapy-induced side effects, as well as to clarify the preventive effects of natural products.
- Preclinical models of chemotherapy and radiotherapy-induced side effects are not sufficiently to verify the prevention of natural products.
- Need an imaged-based high-content screening platform with a protocol to find innovative natural products against chemotherapy and radiotherapy-induced side effects.
- Unknown molecular mechanisms of the attenuations of some natural products on chemotherapy and radiotherapy-induced side effects.
- No strategy of the modulation of gut microbiota by natural products to control chemotherapy and radiotherapy-induced side effects.
- Unknown the biological mechanisms of interaction between chemotherapy drugs and natural products.
- Need to ascertain natural products-evoked toxicity.

## Conclusion

**Figure 1:**
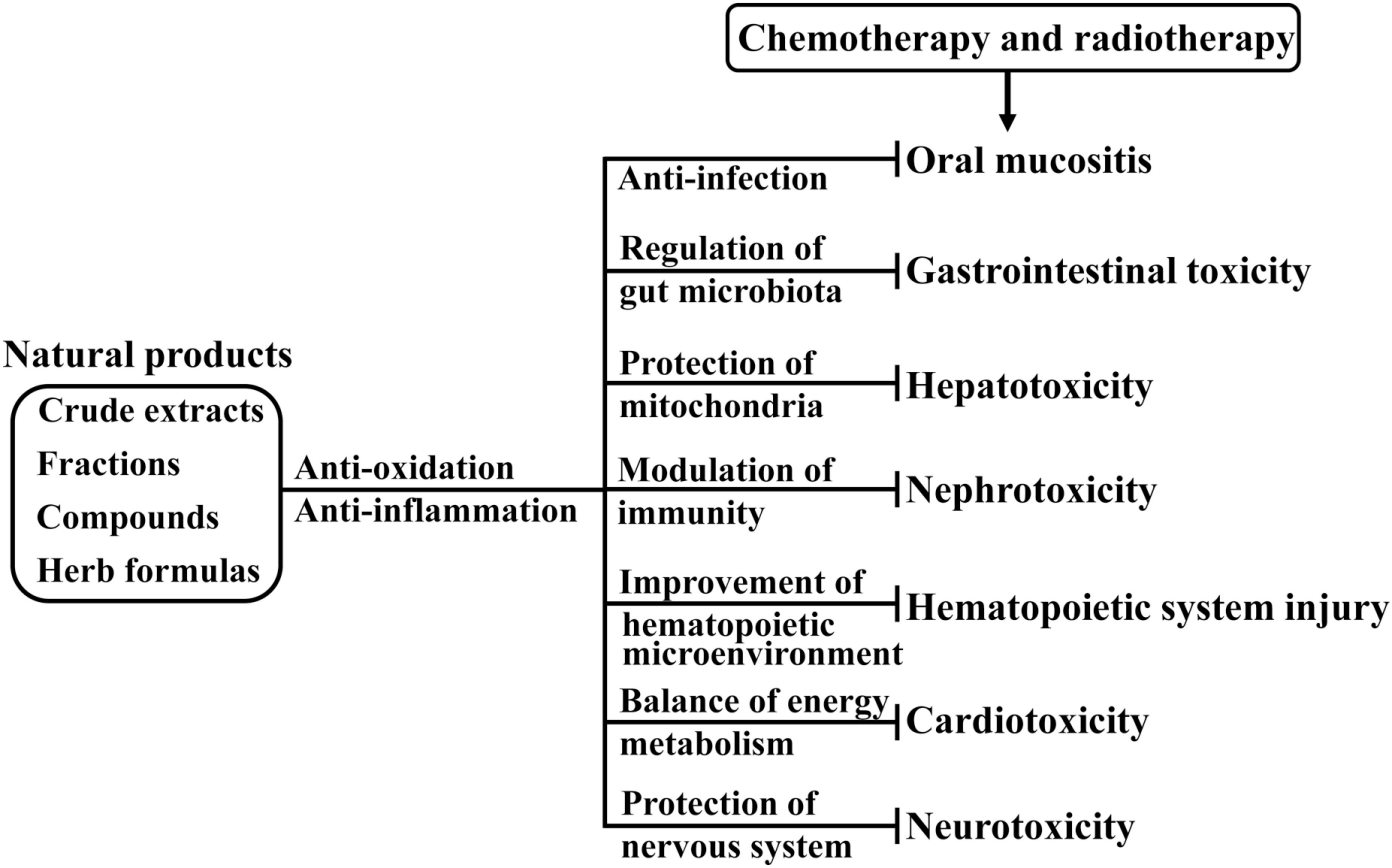
A schematic diagram of the common mechanisms by which natural products reduce chemotherapy and radiotherapy-induced side effects. Natural products including crude extracts, bioactive components-enriched fractions, and pure compounds derived from herbs as well as herbal formulas can effectively reduce chemotherapy and radiotherapy-induced side effects mainly due to their anti-oxidative and anti-inflammatory activities. Moreover, some natural products can attenuate chemotherapy and radiotherapy-induced oral mucositis by anti-infection, mitigate gastrointestinal toxicity by regulating gut microbiota, hepatotoxicity by protecting mitochondria, nephrotoxicity by modulating immunity, hematopoietic system injury by improving hematopoietic microenvironment, cardiotoxicity by balancing energy metabolism, and neurotoxicity by protecting nervous system.

There is the evidence that natural products can reduce chemotherapy and radiotherapy-induced side effects such as oral mucositis, gastrointestinal toxicity, hepatotoxicity, nephrotoxicity, hematopoietic system injury, cardiotoxicity, and neurotoxicity (Figure 1). The summarized data have also highlighted the efficacy of natural dietary supplements to counteract these side effects. Thus, these products including dietary supplements should be used as an alternative therapeutic strategy for the prophylaxis and treatment of chemotherapy and radiotherapy-induced side effects in cancer patients.

However, this issue is still controversial in underlying the efficacy conducted by different analysis and clinical trials in selected cancer patients. The difficulty in the revealing the molecular mechanisms has so far hampered the understanding of the attenuation of chemotherapy and radiotherapy-induced side effects in clinical use of natural products (Anwar et al., 2016).

Of note, new therapies in clinical validation phases are investigating gut microbiota fighting against many diseases, and forming the targets of chemotherapy and radiotherapy-induced toxicity. These studies may help to shed light upon the preventive process of chemotherapy and radiotherapy-induced side effects by natural products and develop the available intervention for cancer patients for the managements.

## Author contributions

LD-K conceived and designed the review. L-DK, Q-YZ, F-XW, and K-KJ performed the literature search. L-DK, F-XW, and Q-YZ analyzed and wrote the paper.

### Conflict of interest statement

The authors declare that the research was conducted in the absence of any commercial or financial relationships that could be construed as a potential conflict of interest.
